# Impact of COVID-19 on psychiatric services and presentations in north-west Edinburgh

**DOI:** 10.1192/bjo.2021.237

**Published:** 2021-06-18

**Authors:** Ritika Devadas, Douglas Murdie, Idris Thomas, Rhona Hannah, Wiktoria Karbowniczek, Tara Kinney, Oscar Lacey-Solymar, Adam Łukaszuk, Seona MacDonald, Sarah McIntosh, Danya Taha, Charles Taylor

**Affiliations:** 1 University of Edinburgh Medical School - 1st year Medical Student; 2 Consultant Psychiatrist, NW CMHT, NHS Lothian

## Abstract

**Aims:**

COVID-19 has had a significant impact on healthcare provision, accessibility and psychiatric presentations. We aim to investigate the impact of the pandemic on psychiatric services and the severity of presentations in Edinburgh, with a particular focus on the North-West Edinburgh Community Mental Health Team (NW CMHT).

**Method:**

Measures of the impact of the pandemic on NW CMHT were identified as referral numbers from primary care and Did Not Attend (DNA) rates. Royal Edinburgh Hospital admissions, detentions under the Mental Health (Care and Treatment) (Scotland) Act 2003 (MHA) and Out of Hours (OOH) contacts were used as proxy measures to explore the severity and urgency of presentations.

Quantitative data focussing on these parameters for patients aged 18–65 years in NW CMHT in 2019 and 2020 were collected from NHS Lothian Analytical Services. OOH data were only available Edinburgh-wide. All data were anonymised in line with NHS Lothian Information Governance Policy.

In order to assess the impact on staff, a questionnaire was created and disseminated, with qualitative data returned anonymously.

**Result:**

Referrals to NW CMHT decreased by 9.3% in 2020 (n = 2164) compared to 2019 (n = 2366). Referrals in April (n = 81) and May (n = 102) 2020 were far below the monthly average across the two years (n = 188).

Appointment numbers were very similar in 2019 (n = 3542) and 2020 (n = 3514). Despite this, DNA and cancellation rates decreased by 3.94% in 2020. Questionnaire results illustrated some of the challenges for staff of working during a pandemic.

Admissions to hospital reduced by 6.8% in 2020 (n = 219 vs n = 235). While MHA detentions in NW Edinburgh increased by only 1.8% (n = 173 vs n = 170), new Compulsory Treatment Orders (CTO) increased by 60%. Furthermore, OOH contacts across Edinburgh increased by 45.2% when compared to 2019.

**Conclusion:**

The COVID-19 pandemic altered the way patients accessed healthcare. Uncertainty of the public in accessing primary care services early in the pandemic may have contributed to reduced referral numbers.

The increase in CTOs is suggestive of severe relapses in previously stable patients or new episodes of illness. The pandemic may have contributed to a reduction in early recognition, and referral, of those with major mental disorders resulting in more protracted or severe illness episodes. The increase in OOH crisis contacts supports such a hypothesis.

Despite what would be expected, DNA and cancellation rates in NW CMHT reduced. The contribution of telemedicine to this warrants further exploration as a means of delivering healthcare in an efficient and accessible way.

